# Histological and Comparative Transcriptome Analyses Provide Insights into Small Intestine Health in Diarrheal Piglets after Infection with *Clostridium Perfringens* Type C

**DOI:** 10.3390/ani9050269

**Published:** 2019-05-23

**Authors:** Zunqiang Yan, Lijuan Cai, Xiaoyu Huang, Wenyang Sun, Shouhu Li, Pengfei Wang, Qiaoli Yang, Tiantuan Jiang, Shuangbao Gun

**Affiliations:** 1 College of Animal Science and Technology, Gansu Agricultural University, Lanzhou 730070, China; yanzunqiang@163.com (Z.Y.); Cailj214@163.com (L.C.); huanghxy100@163.com (X.H.); sun_china@outlook.com (W.S.); wangpf815@163.com (P.W.); yangql0112@163.com (Q.Y.); 2 College of Veterinary Medicine, Gansu Agricultural University, Lanzhou 730070, China; lishouhu88@163.com; 3 Gansu Research Center for Swine Production Engineering and Technology, Lanzhou 730070, China; jiangtt@gsau.edu.cn

**Keywords:** Ileum, Pig, *Clostridium perfringens* type C, Diarrhea

## Abstract

**Simple Summary:**

*Clostridium perfringens* (*C. perfringens*), formerly called *Clostridium welchii*, is a spore-forming pathogenic bacterium. *C. perfringens* type C can produce fatal toxins, which are absorbed by the small intestine into the body causing diarrhea in humans and animals, especially piglets. Each year, diarrhea induced by this pathogen causes significant economic loss to the pig industry worldwide. Nevertheless, the regulatory mechanisms of the duodenum, jejunum, and ileum in piglets challenged by *C. perfringens* type C are poorly understood. This study aimed to identify pathological changes and genes associated with the small intestine in piglets after infection with *C. perfringens* type C. RNA-Sequencing (RNA-Seq), enzyme-linked immunosorbent assay (ELISA), and hematoxylin & eosin (H&E) staining were used to analyze duodenal, jejunal, and ileal tissues. Our results showed that treated piglets were successfully infected with *C. perfringens* type C. These findings will help to elucidate the pathogenicity of piglets infected with this pathogen.

**Abstract:**

*C. perfringens* type C can induce enteritis accompanied by diarrhea and annually causes significant economic losses to the global pig industry. The pathogenic mechanisms of *C. perfringens* type C in pigs are still largely unknown. To investigate this, we challenged seven-day-old piglets with *C. perfringens* type C to cause diarrhea. We performed hematoxylin & eosin (H&E) staining of the small intestine (including duodenum, jejunum, and ileum) and assessed gene expression in the ileal tissue. H&E staining of the duodenum, jejunum, and ileum demonstrated inflammation and edema of the lamina propria and submucosa. A total of 2181 differentially expressed genes (DEGs) were obtained in ileal tissues. Kyoto encyclopedia of genes and genomes (KEGG) pathway analysis of DEGs indicated that the main pathways were enriched in the T cell receptor signaling pathway, NF-kappa B signaling pathway, and (tumor necrosis factor) TNF signaling pathway. These results provide insights into the pathogenicity of *C. perfringens* type C and improve our understanding of host–bacteria interactions.

## 1. Introduction

*Clostridium perfringens* is a gram-positive, anaerobic, spore-forming, and rod-shaped bacterium; it has five different subgroups (A, B, C, D, and E) in the present classification according to four major toxins, namely α (CPA), β (CPB), ε (ETX), and ι (ITX). Moreover, most strains of *C. perfringens* can produce several other toxins including beta 2 (CPB2) and enterotoxin (CPE) [1,2]. These toxins produced by *C. perfringens* are responsible for the process of several diseases, including necrotic enteritis, food poison, diarrhea, and enterogastritis [2,3]. Among the five subgroups, *C. perfringens* type C produces at least two different major toxins (α and β toxin) and mainly causes enteritis characterized by diarrhea in animals, particularly piglets [1,4]. Piglets are susceptible to *C. perfringens* type C and herd mortality rates surpass 30%, which is a high cost to the pig industry [5,6]. In humans, *C. perfringens* type C enters the gastrointestinal tract through the ingestion of contaminated meat (mainly pork) and leads to enteritis necroticans (also known as pigbel or Darmbrand) [4].

Recently, many papers regarding the pathogenesis of *C. perfringens* type C have been published. These studies show that the intestinal tract is the first target organ in *C. perfringens* type C infection and it is the jejunum and ileum in piglets that are mainly damaged by this bacterium [7,8]. Firstly, *C. perfringens* type C colonizes and multiplies in the intestinal tract; next, fatal bacterial toxins are absorbed by the intestinal tract and enter the circulatory system where they may induce a lethal effect [2,9]. RNA-Seq has been used to analyze intestinal transcriptome data from chickens with necrotic enteritis and the results indicate that *C. perfringens* type A infection affects the expression of some immunity genes (such as *IL-4*, *TNFRSF13*, *IL-10*, and *IL-17B*) to resist this bacterium [10,11]. Identification of functional immunity genes in piglets with *C. perfringens* type C could help us further understand the pathogenesis of this infection in order to breed strains of diarrhea-resistant piglets. To date, transcriptome sequencing of the piglet small intestine infected with *C. perfringens* type C has not taken place. Thus, it is necessary to explore the changes that take place in infected piglets and to screen for several immunity genes in an attempt to prevent and control diarrhea through breeding.

In this study, the duodena, jejuna, and ilea were collected and hematoxylin & eosin (H&E) stained for histological analysis. Subsequently, the presence of inflammatory cytokines was detected by enzyme-linked immunosorbent assay (ELISA). Next, we compared the transcriptome profile of mRNA in the ileal tissue of piglets, both non-infected and infected with *C. perfringens* type C, using RNA-Seq. In addition, some of the differentially expressed genes were selected for verification using real-time quantitative PCR (RT-qPCR). This research may accelerate the exploration of candidate genes that can respond to *C. perfringens* type C infection in piglets. Furthermore, it also broadens the understanding of this bacteria-host interaction.

## 2. Materials and Methods 

### 2.1. Ethics Statement

This study was approved by the Committee for Animal Ethics of the College of Animal Science and Technology, Gansu Agricultural University (approval number 2006-398). Experiments were conducted in accordance with the approved guidelines.

### 2.2. Bacterium

The *C. perfringens* type C strain (CVCC 2032) used in this study was purchased from China Veterinary Culture Collection Center (Beijing, China). Cultures were grown in bouillon medium (HopeBio, Qingdao, China) at 37 °C for 16 h with agitation under anaerobic conditions. Cultures were obtained by centrifugation at 3000× *g* for 15 min, washed three times with sterile PBS (pH = 7.2), re-suspended in sterile PBS, and then enumerated depended on ten-fold serial dilutions experiment in yolk plate colony counting by our previous method [12]; density was adjusted to approximately 1 × 10^9^ CFU/mL in preparation for oral challenge.

### 2.3. Animal Experiments

Six seven-day-old piglets were randomly selected; they were healthy, had similar weights, and were not infected with *C. perfringens*, *Escherichia coli,* or *Salmonella* as detected with commercial ELISA kits (Jiancheng Bioengineering Institute, Nanjing, China) by our previously described methods [12]. In this study, these piglets had ad libitum access to antibiotic-free water and feed during the experimental period and each piglet was fed in an open single cage, to avoid cross-infection, at the animal testing ground of Gansu Agricultural University. Three piglets were randomly selected as the control group (CG), while the remaining three piglets were assigned to the treatment group (TG). Each piglet in TG was challenged by an oral gavage of 1 mL 1 × 10^9^ CFU/mL medium once a day for five days, and each piglet in CG was treated with an equal volume of sterile PBS as previously described [12].

### 2.4. Sample Collection

During the experimental period, we collected blood via the anterior vena cava and fecal extracts from all six piglets at 0, 1, 3, and 5 dpi (days post infection). Subsequently, the three piglets with *C. perfringens* type C infection and three piglets without infection were euthanized and duodenal, jejunal and ileal samples were collected. These samples were placed into liquid nitrogen and stored at −80 °C until needed. The tissues were fixed in 10% neutral buffered formalin (NBF) for 24 h and then H&E stained for histological analysis. Fecal extracts were re-suspended in sterile water and then secretory IgA (sIgA) was quantified using an indirect ELISA assay kit (Jiangsu Kete Biological Technology Co., Ltd., Yancheng, China). Blood samples were left to coagulate naturally, and then centrifuged at 2000× *g* for 10 min at 4 °C to obtain the serum, which was used to detect the concentration of pro-inflammatory cytokines (IL-6 and TNF-α) using ELISA assay kits (Jiangsu Kete Biological Technology Co., Ltd., Yancheng, China). All ELISA processes were conducted according to the manufacturer’s instructions.

### 2.5. Clinical Index Records

Rectal temperature was detected daily after the challenge. In addition, all piglets were individually weighed each day. Finally, defecation times and fecal state were recorded. Fecal symptom traits (0 = normal, solid feces, 1 = slight diarrhea, soft and loose feces, 2 = moderate diarrhea, semi-liquid feces, 3 = severe diarrhea, liquid and unformed feces) were judged as previously described [13,14]. Piglets were considered to be diarrheic when the fecal score was at a level of two or greater.

### 2.6. RNA Extraction and Analysis

Total RNA from each sample of ileal tissue was extracted using TRIzol™ reagent (Invitrogen, Carlsbad, CA, USA). The concentration and purity of total RNA were detected using a NanoDrop-2000 spectrophotometer (Thermo Scientific, Waltham, MA, USA). An optical density 260/280 ratio of 1.8 to 2.0 was treated as high quality total RNA. Then, the integrity of RNA was assessed by 1% formaldehyde denaturing gel electrophoresis and qualified total RNA was characterized by an approximate 2:1 ratio of 28S/18S. In addition, total RNA integrity was also assessed using the Bioanalyzer 2100 system (Agilent Technologies, CA, USA) for complementary (cDNA) library construction if RNA integrity number was more than eight.

### 2.7. Library Preparation and Sequencing

According to NEBNext^®^ Ultra™ RNA Library Prep Kit for Illumina^®^ (NEB, Ipswich, MA, USA) instructions, total RNA (3 µg/each ileum sample) was used as an input material for the sequencing library preparations. Firstly, poly-T oligo-attached magnetic beads were used to obtain mRNA from total RNA. Secondly, first strand cDNA synthesis was performed using a random hexamer primer and M-MLV Reverse Transcriptase and second strand cDNA was obtained using DNA polymerase I and RNase H, respectively. Next, adaptors were ligated to blunt ends of the DNA fragments and cDNA fragments (preferentially 150–200 bp lengths) were purified with the AMPure XP system (Beckman Coulter, Beverly, USA). In addition, purified cDNA was treated with a total of 3 µL USER Enzyme at 37 °C for 15 min, followed by 95 °C for 5 min and PCR was performed in buffer containing Phusion High-Fidelity DNA polymerase, Universal PCR primers, and Index (X) Primer. Lastly, PCR products were purified, and library quality was assessed using the Agilent Bioanalyzer 2100 system. Sequencing was performed on an Illumina^®^ Hiseq 4000 instrument (NEB, Ipswich, MA, USA) to generate 150 bp paired-end reads at Novogene Bioinformatics Institute (Beijing, China).

### 2.8. Screening of Differentially Expressed Genes 

High-quality clean reads were aligned to the reference pig genome (*S. scrofa* 10.2) by TopHat [15]. Subsequently, the mapped reads were assembled by Scripture [16] and Cufflinks [17]. Then, the FPKMs (fragments per kilo-base of exon per million fragments mapped) of coding genes were calculated by Cuffdiff [18]. Finally, genes with a corrected *p*-value < 0.05 were considered as differentially expressed.

### 2.9. GO and KEGG Enrichment Analysis

Gene ontology (GO) enrichment and kyoto encyclopedia of genes and genomes (KEGG) pathways of differentially expressed genes were performed using DAVID (https://david.ncifcrf.gov/) [19]. *p*-values < 0.05 were considered significantly enriched GO terms and KEGG pathways.

### 2.10. RT-qPCR Confirmation

Total RNAs of the collected sample tissues were reverse transcribed into cDNA using a PrimeScript™ RT Reagent kit (Takara, Dalian, China) and the cDNA was stored at −20 °C until further analysis. RT-qPCR assays were conducted in a reaction volume of 20 μL (containing 9.5 µL 2 × SYBR Green Realtime PCR Master Mix, 1 µL forward and reverse primers, 1 µL cDNA and 7.5 µL RNase free ddH_2_O) with the Roche LightCycler 480II instrument (Roche, Basel, Switzerland) using the SYBR^®^ Green PCR Master Mix (Takara, Dalian, China). The specific primers of genes were designed in NCBI Primer-BLAST online software and the sequence of primers used for RT-qPCR assays are listed in Table 1. The thermal cycler program included an initial denaturation at 95 °C for 3 min, followed by 40 cycles at 95 °C for 15 s, 58 ± 1 °C for 15 s, and 72 °C for 20 s. Dissociation curves assessed the specificity of PCR products. The relative expression levels of the target genes were calculated with the 2^−∆∆Ct^ method [20] and quantified relative to the *β-actin* gene.

### 2.11. Statistical Analysis

All experimental data were analyzed using SPSS software and represented as mean ± SE. Statistical significance was determined using the two-tailed Student’s *t*-test method.

## 3. Results

### 3.1. Physiological Changes in C. perfringens Type C Challenged Piglets

**Figure 1:**
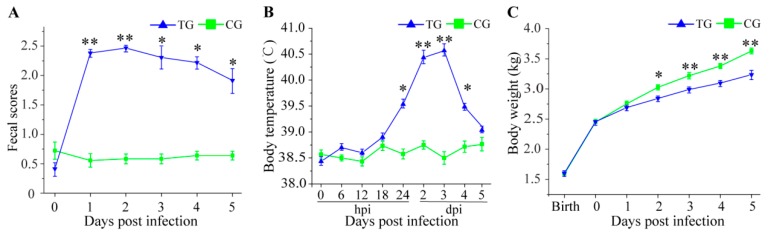
Clinical condition of piglets infected with *C. perfringens* type C. (**A**) Fecal scores from *C. perfringens* type C challenged piglets; (**B**) Rectal temperature from *C. perfringens* type C challenged piglets (hpi represents hours post infection); (**C**) Body weight from *C. perfringens* type C challenged piglets.

Compared to the three piglets in CG, the three piglets in TG displayed extensive and persistent diarrhea (fecal score ≥ 2) within 1 d after the challenge with *C. perfringens* type C (Figure 1A). The TG piglets also exhibited high fever (>40 °C) from 2 dpi to 3 dpi (Figure 1B). Throughout the 5 days, all CG piglets showed normal body weight gain; however, the three TG piglets displayed growth retardation (Figure 1C).

### 3.2. Changes to the Small Intestine in C. perfringens Type C Challenged Piglets

**Figure 2:**
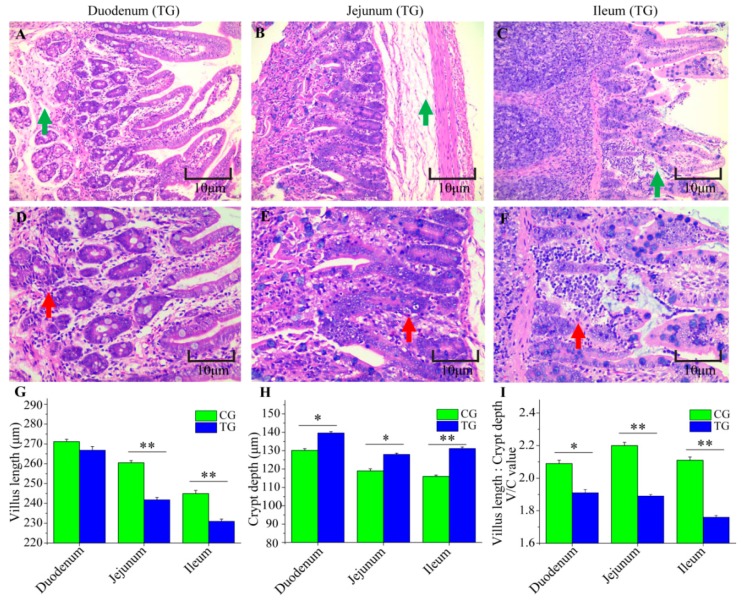
Changes in the small intestine. (**A**) Edema of the lamina propria and submucosa (green arrow); (**B**) Edema of the lamina propria and submucosa (green arrow); (**C**) Edema of the lamina propria (green arrow); (**D**) Inflammatory cell infiltration (red arrow); (**E**) Infiltration with neutrophil granulocytes (red arrow); (**F**) Inflammatory cell infiltration (red arrow); (**G**)Villus height; (**H**) Crypt depth; (**I**) The ratio of villus width/crypt depth. Asterisk above bars indicates a significant difference (* *p* < 0.05, ** *p* < 0.01). Data are shown as mean ± SE of thirty replicates.

**Figure 3:**
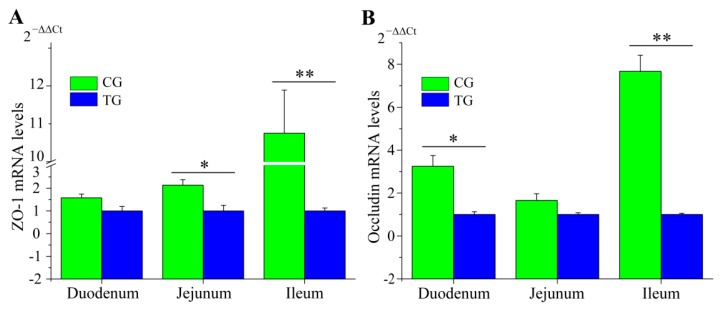
Tight junction protein genes *ZO-1* (**A**) and *Occludin* (**B**) mRNA levels in the small intestinal by RT-qPCR. Data are presented as mean ± SE of three replicates (* *p* < 0.05; ** *p* < 0.01).

The morphology of the duodena, jejuna, and ilea from CG and TG were examined (Figure 2A–I). Compared with CG, the tissues from TG piglets were abnormal; edema of the lamina propria and submucosa were found in duodenal and jejunal tissues (Figure 2A,B); edema of the lamina propria was also was found in the ileal tissue (Figure 2C). Furthermore, there was inflammatory cell infiltration in the duodenal and ileal tissues (Figure 2D,F) and infiltration of small amounts of neutrophile granulocytes appeared in the jejuna (Figure 2E). Additionally, in the jejunal and ileal tissues, villi length was significantly lower in TG than that in CG (Figure 2G). Between CG and TG, significant differences were observed for crypt depth, which was higher in the duodenal, jejunal and ileal tissue after infection (Figure 2H). The ratio of villus width to crypt depth in the three intestinal tissues of TG was lower than that in CG (Figure 2I). To assess the integrity of the intestinal membrane, we detected the expression level of *ZO-1* and *Occludin* genes using RT-qPCR. Compared to piglets in CG, two genes were significantly decreased in TG, especially in the ileum (Figure 3A,B). 

### 3.3. Dynamic Change of Inflammatory Cytokines and sIgA

**Figure 4:**
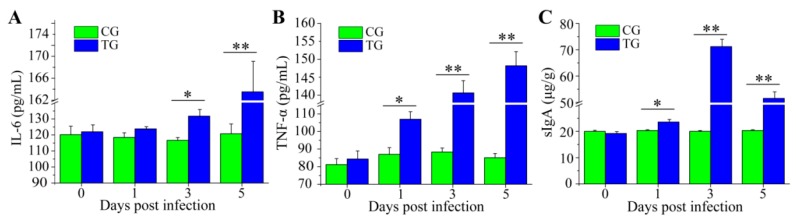
Immune response to *C. perfringens* type C infection. Serum concentration of pro-inflammatory cytokines IL-6 (**A**), TNF-α (**B**), and the fecal levels of sIgA (**C**) at 0, 1, 3, and 5 dpi were evaluated by ELISA. Each sample was assayed in three replicates. Values are presented as mean ± SE (* *p* < 0.05; ** *p* < 0.01).

In the gut lumen, sIgA is treated as the first line of defense in protecting the intestinal epithelium from pathogens. Fecal sIgA in TG and CG was examined throughout the experiment. The results indicated that the sIgA levels in TG were not different at 0 dpi and gradually increased at 1 dpi. Obviously, piglets in TG had significantly higher fecal sIgA levels at 3 dpi and 5 dpi as compared to CG (Figure 4C). After exposure to *C. perfringens* type C, the content of pro-inflammation cytokines IL-6 and TNF-α was increased from 1 dpi to 5 dpi (Figure 4A,B).

### 3.4. DEGs of Ileum after Infection

**Figure 5:**
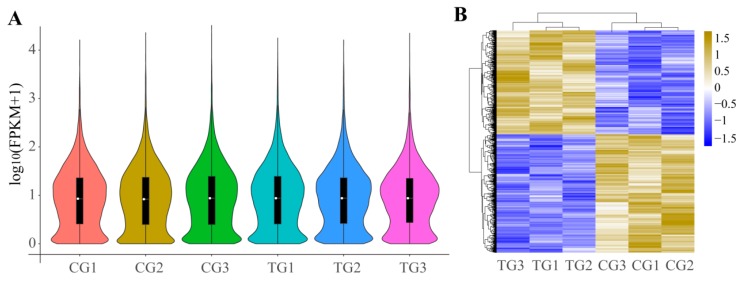
(**A**) Expression level indicated by log10 (FPKM + 1) in the mRNAs between control piglets (CG) and treatment group (TG); (**B**) Clustered heatmap of the differentially expressed mRNAs in paired samples of CG and TG. Rows represent mRNAs while columns represent different treated samples.

We explored the variation in DEGs using pairwise comparisons between CG and TG. No differences were observed in gene expression between CG and TG (Figure 5A). A total of 2181 DEGs (including 1021 up-regulated and 1160 down-regulated genes) were found. In addition, the heatmap displayed all DEGs between the two groups and indicated that three samples in CG or TG had similar expression patterns (Figure 5B).

### 3.5. Functional Analysis of DEGs

**Figure 6:**
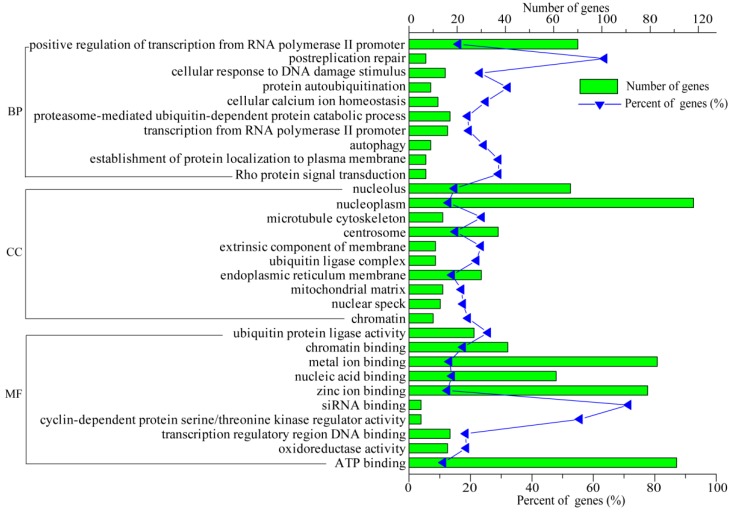
Gene ontology (GO) terms were classified into cellular component (CC), molecular function (MF), and biological process (BP). The top 30 GO terms are selected according to a *p*-value < 0.05.

To explore DEGs function, GO analysis was performed on three different aspects, including biological process (BP), molecular function (MF), and cellular components (CC). In order to identify the potential and useful genes for deep investigation, the top 30 GO terms were screened (*p*-value < 0.05) and are listed in Figure 6. GO analysis showed that the DEGs between the two groups were enriched in the protein autoubiquitination and cellular calcium ion homeostasis in BP; in the microtubule cytoskeleton, extrinsic component of the membrane, and endoplasmic reticulum membrane of the CC; and in the nucleic acid binding and transcription regulatory region DNA binding in MF (Figure 6).

**Figure 7:**
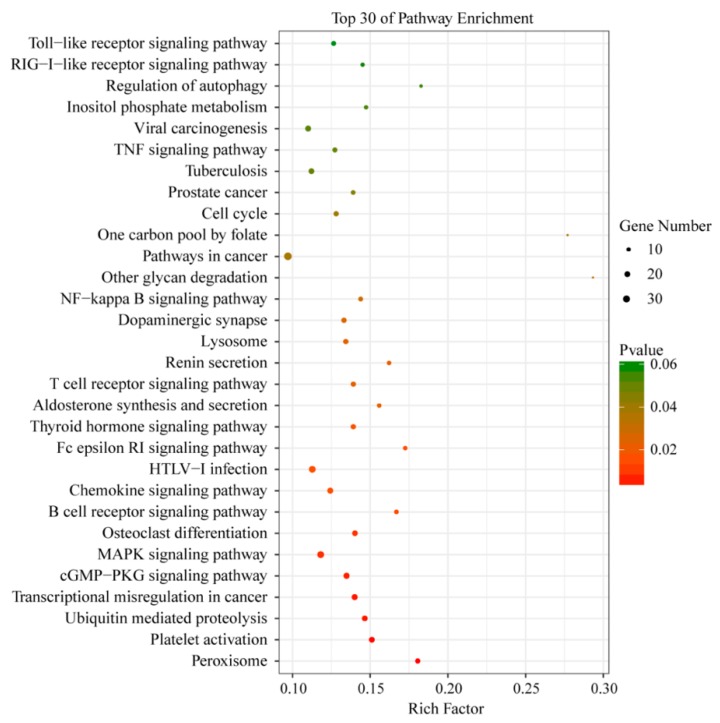
KEGG pathways of the differentially expressed genes. Rich factor is the ratio of the number of genes located in the KEGG pathway to the total number of genes in the KEGG pathway. The top 30 KEGG pathways are listed according to a *p*-value < 0.05.

To further define DEGs function in the ileum after *C. perfringens* type C infection, the DAVID database was used to analyze these DEGs. The top 30 enriched KEGG pathways of the DEGs are listed according to a *p*-value < 0.05 in Figure 7. Several functional classifications were selected to potentially play important roles related to *C. perfringens* type C infection, including B cell receptor signaling pathway, T cell receptor signaling pathway, NF-kappa B signaling pathway, TNF signaling pathway, and Toll-like receptor signaling pathway.

### 3.6. RT-qPCR Validation of RNA-Seq Data

**Figure 8:**
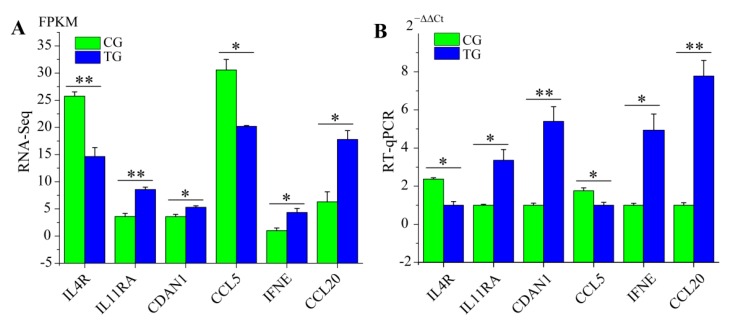
Verification of RT-qPCR for some differentially expressed genes. (**A**) RNA-Seq results. (**B**) RT-qPCR results. The results are shown as the mean ± SE of three replicates. (* *p* < 0.05; ** *p* < 0.01).

To evaluate the DEGs identified by the transcriptome sequencing data, we detected the expression levels of six immune-related genes, which were mainly enriched in host immune defense responses against *C. perfringens* type C infection. These genes included *IL4R* (Interleukin 4 receptor), *IL11RA* (Interleukin 11 receptor subunit alpha), *CDAN1* (Codanin 1), *CCL5* (C-C motif chemokine ligand 5), *IFNE* (Interferon epsilon), and *CCL20* (C-C motif chemokine ligand 20). The RT-qPCR results were consistent with the results of RNA-Seq analysis, which demonstrated that the RNA-Seq data was highly reliable and accurate in this study (Figure 8).

## 4. Discussion

*C. perfringens* type C causes diarrhea characterized by high morbidity and mortality in pigs, especially newborn and suckling piglets [4]. The occurrence of diarrhea is mainly induced by contact with infected pigs or contaminated food. In recent years, *C. perfringens* type C has become one of the most widespread bacterial infections in the global pig industry and has resulted in great economic loss [4]. To date, RNA-Seq technology has been used to disclose the biological processes and the development of some diseases, including necrotic enteritis [11,23,24], diarrhea [25,26], arthritis [27,28], and even various cancers [29,30]. Additionally, some studies have explored transcriptome sequences of different tissues (including spleen and small intestine) in *Escherichia coli* [31,32] and *Streptococcus suis* type 2 [33] infected pigs, giving a huge amount of basic data for illustrating the mechanism of pathogenic bacteria in pigs. A greater understanding of piglet response to *C. perfringens* type C infection may help us prevent and control diarrhea caused by this bacterium. However, knowledge of the mechanics of piglet response to *C. perfringens* type C is still limited. Therefore, in this study, we analyzed changes in the small intestine and investigated the transcriptome of the ileum in normal piglets and in piglets at 5 dpi with *C. perfringens* type C to accelerate investigations into the molecular events of infection. In short, results from our current study indicated that transcriptome analysis of RNA-Seq data may assist in the understanding of the precise mechanisms of diarrhea caused by *C. perfringens* type C in piglets.

It is known that pigs challenged by various pathogenic bacteria display diarrhea, growth retardation, and high fever [22,34,35]. In our study, we also found that piglets in TG exhibited diarrhea and body weight loss (Figure 1A,C), which suggested that *C. perfringens* type C and its fatal toxins could impair the integrity of the intestinal barrier and then caused diarrhea; persistent diarrhea then led to body weight loss. *C. perfringens* type C and its toxins may trigger the innate immune response, causing an obvious high fever in piglets (Figure 1B). Previous reports show that the main target organ for *C. perfringens* type C is the small intestine in animals [4,36]. H&E staining was used to determine whether there were pathological changes in the small intestine of infected piglets in the current study and revealed the principle lesions of inflammation and edema (Figure 2A–F). This result was consistent with our previous results in the small intestine of piglets challenged by *C. perfringens* type C [12]. Villi of the small intestine play an important role in absorbing nutrients for animal growth and development [37,38]. Moreover, previous studies report that the infection may reduce the mitotic potential of the villi, causing a decrease in villi height and an increase in villi depth [22,39,40]. Indeed, piglets in TG had lower villi and larger crypts in the small intestine as compared with CG (Figure 2G–I), which suggested that *C. perfringens* type C induced enteric changes.

Tight junction proteins (including ZO-1 and Occludin) seal the paracellular space between epithelial cells for maintaining the integrity of the intestinal barrier [41]. The integrated tight junction barrier plays key roles in ion transport, inflammation during intestinal epithelial responses to enteric pathogenic bacteria, such as enterotoxigenic *E. coli*, and *Salmonella* [42,43,44]. Moreover, damage to the integrity of the intestinal barrier increases the risk of infection in animals [45,46]. In this study, we observed that the expression level of *ZO-1* and *Occludin* were decreased after *C. perfringens* type C infection (Figure 3A,B). These results suggested that *C. perfringens* type C can break the integrity of the intestinal barrier by destabilizing and dissociating the tight junction proteins (ZO-1 and Occludin) in the small intestine. We speculated that these changes of the small intestine could lead to diarrhea, which would, in turn, lead to growth retardation.

Proinflammatory cytokines (such as IL-6 and TNF-α) play an important role in the regulation of enteric pathogenic bacteria and the concentration of these cytokines in the serum of animals is usually increased after enteric pathogenic bacterial infection [22,47,48]. IL-6 plays a key role in stimulating B cell proliferation and T cell proliferation, and antibody production [49,50]. TNF-α is associated with cell-mediated immune response and confers immunity against harmful agents including bacteria, viruses, and even tumor cells [51,52]. In our study, compared to the piglets in CG, the levels of IL-6 and TNF-α in serum were increased in TG piglets (Figure 4A,B). The increased concentration of IL-6 and TNF-α were beneficial for enhancing immunity and thus resisting *C. perfringens* type C. sIgA serves as an important indicator of mucosal immunology, it plays a vital role in protecting the epithelium from pathogens, and fecal sIgA levels are related to microorganism infection [53,54]. In our study, we observed that the concentration of fecal sIgA in CG was lower than that in TG (Figure 4C). These results indicated that the host immune system response was activated after piglets were infected with *C. perfringens* type C.

We also screened and verified differentially expressed transcripts in *C. perfringens* type C-infected piglet’s ileal tissues using RNA-Seq. GO terms and KEGG signaling pathway analyses were performed to confirm differentially expressed gene function. By comparing the transcriptome data of the ileum from TG and CG, the results showed that most of the DEGs were involved in many immunological responses of the KEGG pathways, including the NF-kappa B signaling pathway, TNF signaling pathway, and Toll-like receptor signaling pathway (Figure 7). These KEGG pathways are also enriched in the small intestine tissue of chickens infected with *C. perfringens* type A [10,11]. In our previous study, we also found that these KEGG pathways were enriched in piglets challenged with *C. perfringens* type C infection [12]. The latter results suggest that these KEGG pathways play crucial roles in anti-*C. perfringens* type C response and piglet defense during *C. perfringens* type C infection. Additionally, we detected the expression levels of six differentially expressed genes in piglet spleens between CG and TG. IFNE belongs to a type I IFN and plays key roles in resisting pathogenic microorganisms [55,56]. After *C. perfringens* type C infection, the expression of *IFNE* was significantly up-regulated. Studies have found that CCL20 is a chemokine with antimicrobial activity [57,58]. We observed that the expression level of *CCL20* was higher in TG than that in CG. This indicated that up-regulated *CCL20* may inhibit *C. perfringens* type C invasion and reproduction in piglets. In summary, these immune genes (such as *IFNE* and *CCL20*) may play an important role via KEGG pathways in the response of piglets to *C. perfringens* type C infection.

## 5. Conclusions

In this study, we compared the fecal scores, rectal temperature, body weight, pathological change of the small intestine, and pro-inflammation cytokines (IL-6 and TNF-α) between control piglets (CG) and piglets challenged with *C. perfringens* type C (TG). Subsequently, we used RNA-Seq to investigate the genetic profile of the ileum in piglets. The study revealed a set of candidate genes (such as *IFNE* and *CCL20*) that may contribute to *C. perfringens* type C infection in piglets. This study offers information towards a deeper understanding of the immune response of piglets to *C. perfringens* type C infection.

## Figures and Tables

**Table 1:** Primers used for real-time quantitative PCR analysis

Gene	Accession Number	Sequence (5′-3′)	Product Size (bp)	Reference
*Occludin*	NM_001163647.2	TCCTGGGTGTGATGGTGTTC	144	[21]
		CGTAGAGTCCAGTCACCGCA		
*ZO-1*	XM_021098827.1	TGAGTTTGATAGTGGCGTTG	298	[22]
		TGGGAGGATGCTGTTGTC		
*IL4R*	NM_214340.1	GTGGCCCATCTGCCTATCC	161	
		CTGAGCCTGCTCTGTTCTCG		
*IL11RA*	XM_021064672.1	CCGCAACAGTGTCGCTAGT	201	
		CCACAGAGACCTTCCCCAAA		
*CDAN1*	XM_021097154.1	TTTTGAGAAGGGCTTGGGCA	160	
		ATCCGGAGTCTCACCCAAGA		
*CCL5*	NM_001129946.1	TGCTTCTTGCTCTTGTCCCA	189	
		GTGCCAAGGGTCCAAAGTTC		
*IFNE*	NM_001105310.1	GTGTCTGCCACACCGGAAAA	160	
		GTGGCTTTCCTCCCAACCAT		
*CCL20*	XM_005672261.3	ATCTGGGTGAAACAAGCCGT	185	
		TGGACAAGTCCAAAGAGGCA		
*β-actin*	XM_003124280.5	AGGCGGACTGTTAGTTGCAT	187	[12]
		TGTCACCTTCACCGTTCCAG		
